# Global Genome Comparative Analysis Reveals Insights of Resistome and Life-Style Adaptation of *Pseudomonas putida* Strain T2-2 in Oral Cavity

**DOI:** 10.1155/2014/930727

**Published:** 2014-11-10

**Authors:** Xin Yue Chan, Kah Ooi Chua, Kah Yan How, Wai-Fong Yin, Kok-Gan Chan

**Affiliations:** Division of Genetics and Molecular Biology, Institute of Biological Sciences, Faculty of Science, University of Malaya, 50603 Kuala Lumpur, Malaysia

## Abstract

Most *Pseudomonas putida* strains are environmental microorganisms exhibiting a wide range of metabolic capability but certain strains have been reported as rare opportunistic pathogens and some emerged as multidrug resistant *P. putida*. This study aimed to assess the drug resistance profile of, via whole genome analysis, *P. putida* strain T2-2 isolated from oral cavity. At the same time, we also compared the nonenvironmental strain with environmentally isolated *P. putida*. *In silico* comparative genome analysis with available reference strains of *P. putida* shows that T2-2 has lesser gene counts on carbohydrate and aromatic compounds metabolisms, which suggested its little versatility. The detection of its *edd* gene also suggested T2-2's catabolism of glucose via ED pathway instead of EMP pathway. On the other hand, its drug resistance profile was observed via *in silico* gene prediction and most of the genes found were in agreement with drug-susceptibility testing in laboratory by automated VITEK 2. In addition, the finding of putative genes of multidrug resistance efflux pump and ATP-binding cassette transporters in this strain suggests a multidrug resistant phenotype. In summary, it is believed that multiple metabolic characteristics and drug resistance in *P. putida* strain T2-2 helped in its survival in human oral cavity.

## 1. Introduction

*Pseudomonas putida* are aerobic Gram-negative, nonlactose-fermenting, and oxidase-positive bacteria [1]. As a member of the fluorescent group of* Pseudomonas*,* P. putida* produces pyoverdin, a yellow-green pigment that fluoresces under UV light and is grouped as nonphytopathogenic, nonnecrogenic strains together with* P. aeruginosa* and* P. fluorescens *[2, 3]. Like the other members in this genus, the species exhibits a diverse metabolic capability making it famous to be able to inhabit a wide range of environments including soil that is contaminated with aromatic and toxic compounds, freshwater, and surfaces of living organisms [4]. To date,* P. putida* has been described with ability to degrade nicotine [5], benzoate [6], phenol [7], toluene [8], and alkane [9].

In spite of the fact that it is ubiquitous in various environments, isolation of* P. putida* from clinical specimen is rare. However, cases of* P. putida* infections had been reported and occurrence of the species in catheter caused outbreak of nosocomial infections which contributed to bacteremia in adult patients [10] as well as neonates [11]. In addition, in a review on* P. putida* infections of marine military personnel, a majority of the infections reported were cases of bacteremia, urinary tract infections, and pneumonia as well as uncommon infections on skins and soft tissues, peritoneum, central nervous system, ears, joints, and cornea [3]. These findings are alarming as* P. putida* is becoming a threat to public health care. This is especially critical when a* P. putida* strain HB3267 had been reported as multidrug resistant recently, which resists beta-lactams antibiotics, fluoroquinolone, macrolides, aminoglycosides, polymyxin, and polyketide [12].

As a species commonly found in the environment, association of* P. putida* as commensal organism in human is rarely described. A strain of the species,* P. putida* T2-2, had been isolated from a healthy individual's tongue surface and reported for its quorum sensing activity [13]. As there is reason to believe that the species is an opportunistic pathogen with ability to cause life-threatening diseases in human, it raises our concern about the emergence of the multidrug resistant strains. Consequently, in order to assess the difference in genomic profile and antibiotic resistance of clinically isolated* P. putida* and those obtained from the environment,* P. putida *strain T2-2 (hereafter referred to as T2-2) that was previously identified as commensal organism on a healthy individual's tongue surface was employed as reference culture for a comparative genome analysis. In this experiment, we whole-genome sequenced this isolate using next generation sequencing and performed genome analysis on the whole genome sequences of T2-2. We then furthered our study and reported the findings from comparative analysis between the draft genome of T2-2 and available whole genome of several environmental* P. putida* strains in the database.

## 2. Materials and Methods

### 2.1. Isolation of* P. putida* Strain T2-2 and Genomic DNA Extraction

Isolation and characterization of bacteria strain T2-2 have been described by Chen et al., 2013 [13]. Strain T2-2 was maintained and cultured in LB medium. The genomic DNA of strain T2-2 was extracted and purified with QIAamp DNA minikit (Qiagen). The extracted DNA was examined by NanoDrop Spectrophotometer (Thermo Scientific) for quality and quantified by Qubit 2.0 fluorometer (Life Technologies) using dsDNA High Sensitivity Assay Kit (Invitrogen). Extracted genomic DNA was stored in −20°C.

### 2.2. Library Preparation and Whole Genome Sequencing

The sequencing library was prepared using TruSeq DNA Sample Preparation Kit (v2) (Illumina) and subjected to next generation sequencing (NGS) on HiSeq 2000 (Illumina) platform [14].

### 2.3. Reference Genome Assembly

Closely related* P. putida *strains were determined from dendrogram based on genomic BLAST from National Center for Biotechnology Information (NCBI) [15]. Complete genomes of closely related* P. putida *and RAST available strains were downloaded from NCBI as reference genomes. Reference genome assembly was performed with CLC Genomic Workbench (v6.0.5) [16].

### 2.4. Genome Assembly and Annotation

The raw reads generated were trimmed and* de novo* assembled with CLC Genomic Workbench (v6.0.5). Contigs with minimal length of 200 bp and coverage of 30-fold were selected for subsequent analysis. The genes were annotated with RAST (rapid annotation using subsystem technology) (v4) [17]. The number of rRNA genes was determined with RNAmmer [18] while tRNA genes were predicted with RAST [17].

### 2.5. Comparative Genome Analysis

The draft genome of T2-2 was used as reference genome and compared with genome of* P. putida* GB-1 (hereafter referred to as GB-1),* P. putida* F1 (hereafter referred to as F1), and* P. putida* W619 (hereafter referred to as W619) available in the RAST database.

### 2.6. Antibiotic Susceptibility Testing

McFarland 0.5 standard in 0.45% sodium chloride (NaCl) was used as standard in measuring the turbidity of cell suspension. Bacteria turbidity was adjusted to 0.5–0.63 in 3 mL of 0.45% NaCl (AirLife) and measured with densitometer (bioMerieux) [19]. AST-GN83 card was read by Vitek 2 (bioMerieux) and the antibiogram was generated by the software [20]. The antimicrobials tested were ampicillin, amoxicillin/clavulanic acid, ampicillin/sulbactam, piperacillin/tazobactam, cefazolin, cefuroxime, cefuroxime axetil, cefoxitin, cefotaxime, ceftazidime, ceftriaxone, cefepime, aztreonam, meropenem, amikacin, gentamicin, ciprofloxacin, nitrofurantoin, and trimethoprim/sulfamethoxazole. The result was analyzed with VITEK 2 Advanced Expert System.

### 2.7. Molecular Detection of 6-Phosphogluconate Dehydratase Gene (*edd*) by PCR

The* edd *gene was amplified using forward primer: 5′-ACCAGCCTTACTTGCACTTC-3′ and reverse primer: 5′-CCATCACTTCCATCACCAACT-3′.* Pseudomonas putida *S13 (hereafter referred to as S13) was employed as positive control. The PCR condition was as follows: denaturation at 95°C for 2.5 min; denaturation at 95°C for 40 s, annealing at 54.5°C for 40 s, and extension at 72°C for 30 s repeated for 35 cycles. The final elongation step was at 72°C for 3 min. PCR amplification was performed in 20 *μ*L volume with Bio-Rad T100 Thermal Cycler. The amplified DNA was purified and the gene sequence was confirmed by Sanger sequencing.

### 2.8. Gene Alignment and Comparison

Amino acid sequences of* edd *genes from* P*.* putida *T2-2 and S13 were generated based on their DNA sequences using MEGA (v5.2.2). Their DNA sequences were aligned with reference sequences from NCBI [21]. Dot plots of these aligned amino acids and DNA sequences were generated using LAST [22].

## 3. Results and Discussion

High quality reads from strain T2-2 were mapped against reference genome* P*.* putida *strains W619, HB3267, GB-1, and F1 and the details were listed in Table 1. The genome of* P*.* putida *strain T2-2 was mapped with reference genomes with at least 80 X coverage (Table 1). However, based on the reference mapping result, less than 70% of the reads were mapped to the reference genomes. This suggested that genomic differences with probable insertions or deletions occurred in the genome of strain T2-2.

Owing to the low percentage of reads that were mapped to the reference genome,* de novo *assembly was performed to acquire more information of the genome of* P*.* putida *T2-2. The draft genome from the* de novo *assembly resulted in 389 contigs with a genome size of 5.5 Mbp. The G + C content of the genome is 62.57% with an N50 of 16.3 kb and a total of 5,053 coding DNA sequences (CDSs) were predicted by RAST, 4 copies of rRNA operons as predicted by RNAmmer and 74 copies of tRNA genes as predicted by RAST. The draft genome is deposited into DDBJ/EMBL/GenBank under the accession JALX00000000. The version described in this paper is the first version, JALX01000000.

**Figure 1:**
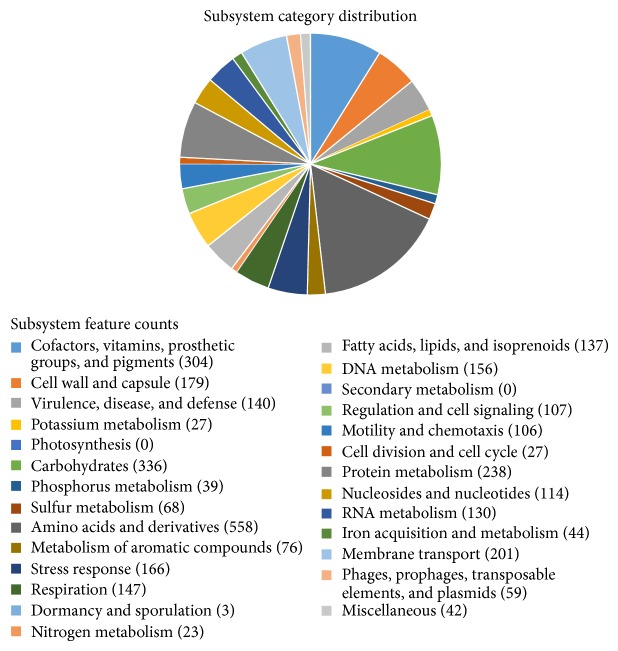
Subsystem distribution of* P. putida* T2-2 genome. Whole genome sequence was annotated and the subsystem distribution was generated from RAST.

From RAST annotation, the closest neighbours of T2-2 were* P. putida *B6-2 (score 545), followed by* P. putida* F1 (score 502) and* P. putida* KT2440 and* P. putida* GB-1 (score 494). The present whole genome analysis of bacterial identification was in agreement with the previous report that relied on phylogenetics analysis of its 16S rDNA (Accession number: HQ907954) which clearly identified T2-2 as* P. putida *[13]. The 5,053 CDSs predicted by RAST were divided into subsystems and shown in Figure 1. All essential genes identified in T2-2 were present in the genome of T2-2 and the genes functions were assigned to nutrients metabolism, respiration, and cell division.

The summary of T2-2 draft genome and other selected genomes of environmental* P. putida* strains is shown in Table 2. Comparison on their subsystem distributions revealed the nonenvironmental lifestyle of T2-2 with lesser gene count (336) on carbohydrates metabolism as compared to GB-1 (445), F1 (473), and W619 (410). With regard to one-carbon (C1) compounds metabolism, no CDS for serine-glyoxylate cycle that was responsible for C1-compounds metabolism was detected by RAST annotation in T2-2. Similar finding was obtained for W619 that was isolated from rhizosphere of plants as endophyte [23]. It is believed that serine-glyoxylate cycle enables GB-1 and F1 to assimilate C1 compounds in the environment such as rhizoplane [24].

**Figure 2:**
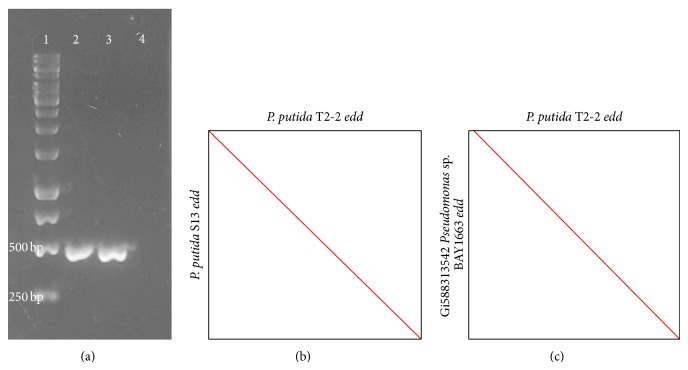
(a) Agarose gel image for 1: 1 kb DNA ladder; 2: PCR product* edd* gene in* P. putida *T2-2; 3: PCR product* edd* gene in* P. putida* S13 which serves as positive control, and 4: negative control. (b) Dot plot from DNA sequence of* edd* gene of* P. putida* T2-2 against* P. putida* S13. (c) Dot plot from amino acid sequence of* edd *gene of* P. putida* T2-2 against reference gene* Pseudomonas* strain BAY1663.

Besides, the glucose metabolism in* P*.* putida *T2-2 was studied and its ability to consume glucose was observed (data not shown). Due to the absence of 6-phosphofructokinase for Embden-Meyerhof-Parnas (EMP) route, glucose metabolism in* P. putida* is mainly through Entner-Doudoroff (ED) pathway [25]. In this study, the PCR-amplified 471 bp DNA fragment was predicted to be the* edd *gene that codes for 6-phosphogluconate dehydratase (Figure 2(a)) in T2-2. Similar PCR product was obtained from the same species* P. putida *strain S13 (Figures 2(a) and 2(b)). Based on the gene alignment and comparison from LAST, it showed high similarity with 6-phosphogluconate dehydratase of the reference genome* Pseudomonas* sp. BAY1663 (Accession number: gi588313542) (Figure 2(c)). In both ED and EMP pathways, the hexose sugars are phosphorylated and subsequently cleaved by aldolase enzymes into two three-carbon products [26]. However, ED pathway was distinct from EM pathway by two key enzymes which were the 6-phosphogluconate dehydratase (Edd) and KDPG aldolase (Eda) [26].

Analysis on CDS under the subsystem of virulence, disease, and defence in* P*.* putida *strain T2-2 revealed the presence of genes responsible for iron acquisition which is an important aspect of virulence due to the scarcity of iron available for bacteria in the human body [27]. Besides, CDS encoding for resistance and tolerance to heavy metals including copper, arsenic, chromium, and cobalt-zinc-cadmium were detected from strain T2-2 as well as W619, HB3267, F1, and GB-1.

In addition, CDSs responsible for lysozyme inhibition, beta-lactamases, and resistance towards antibiotic fluoroquinolones are present in their genomes as well. This prediction in T2-2 was supported by antibiogram with higher than expected minimal inhibitory concentrations (MICs) readings in strain T2-2 when tested with *β*-lactam antibiotics including ampicillin, amoxicillin, sulbactam, cefazolin, cefuroxime, cefuroxime axetil, cefoxitin, and fluoroquinolones compound, nitrofurantoin (Table 3). Even though* P. putida *infection in human rarely happens, we should be aware of the emergence of multidrug resistant strains as it had been reported as the causal agent of nosocomial infection, urinary tract infection, and other diseases [3, 28, 29].

However, a clear, distinctive characteristic of the T2-2 strain is its possession of a group of CDSs encoding for resistance-nodulation-division (RND) multidrug efflux pumps that are not present in the genomes of the other three* P. putida* strains selected from RAST (see Supplementary Table 1 in Supplementary Material available online at http://dx.doi.org/10.1155/2014/930727). These RND pumps function as a tripartite protein complex that consists of the outer membrane protein, membrane fusion protein (the periplasmic component), and inner membrane component [30]. The complex forms a channel across the cell membrane, enabling the pumping of molecules (such as antibiotic) across the bacterial periplasm and the outer membrane into the external medium efficiently as this process is driven by energy derived from proton gradient [30]. In* Pseudomonas aeruginosa,* the RND pumps elevate its drug resistance by causing a slower accumulation of drug inside the cell [31].

**Figure 3:**
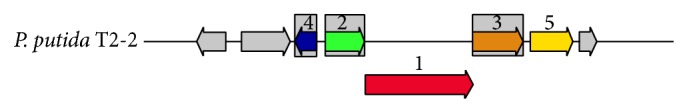
Genes organization of multidrug resistance efflux pumps identified by RAST annotation of T2-2 genome. (1) RND efflux system, inner membrane transporter, CmeB; (2) membrane fusion protein of RND family multidrug efflux pump; (3) RND efflux system, outer membrane lipoprotein, CmeC; (4) transcription repressor of multidrug efflux pump acrAB operon, TetR(AcR) family.

The schematic diagram of RND genes organization encoding the mentioned system in T2-2 is shown in Figure 3. According to RAST annotation, the CDSs encode proteins including membrane fusion protein CmeA, inner membrane transporter CmeB, and outer membrane lipoprotein CmeC (Figure 3). The proteins CmeABC have been reported as multidrug efflux system in* Campylobacter jejuni *contributing to resistance towards a wide range of antibiotics, heavy metals, bile salt, and other antimicrobial agents [32]. Interestingly, the presence of the system as a complete tripartite protein complex is revealed by draft genome analysis in T2-2 supporting resistance of this strain towards the therapeutic antimicrobial agents [30].

Besides, a wide range of adenosine triphosphate (ATP) binding cassette (ABC) transporters has been discovered in genome of T2-2, which are not noticed in the other three reference genomes of environmental strains. These include the ABC transporters for alkyl phosphate, oligopeptide, branched amino acids, and dipeptide. These ABC transporters play an important role in the active transport systems of the archaea, eubacteria and eukaryotic cells [33, 34]. They are responsible for importing and exporting wide variety of substrates in and out of the bacteria cell, which include carbohydrates and sugars, amino acids, and peptides, as well as metal ions [35]. The presence of the ABC transporters in T2-2 is believed to assist this bacterium in uptake of partially digested solutes in the environment of oral cavity, making it adapt better in the habitat.

However, coupling of ABC transporters and multidrug efflux pump encourages the emergence of multidrug resistant strain as the energy (ATP) supplied by ABC transporter enables the active translocation of antibiotic molecules [36]. This might make T2-2 able to survive or even counter-measure the antibacterial drugs it encountered in colonization of the oral cavity.

## 4. Conclusions

The comparative analysis of draft genome of* P. putida *T2-2 revealed lesser gene counts in metabolism of carbohydrates and aromatic compounds as compared to selected environmental strains. In order to adapt to the human oral cavity, this strain possesses extra putative ABC transporter systems for various substrates which might help in its survival in the oral cavity. Moreover, the putative presence of additional multidrug efflux pump genes in the genome reveals its possible multidrug resistant nature.

## Supplementary Material

Pseudomonas putida strain T2-2 carries genes that encode for multidrug resistance efflux pump as well as multidrug and toxin extrusion family efflux pump. These genes were not found within the environmental Pseudomonas (reference genome from RAST).The presence of these efflux pumps protect it from the antibiotic and antimicrobial compounds which were presence in the human oral cavity.

## Figures and Tables

**Table 1:** Summary of reference genome mapping of *P*. *putida *T2-2 against the complete genome of *P*. *putida *W619, *P*. *putida *HB3267, *P*. *putida *F1, and *P*. *putida *GB-1.

Reference genome	GenBank accession number	Consensus length, (Mbp)	Average coverage	Reads map to reference genome, %	Bases map to reference genome, %
*P. putida* W619	CP000949.1	5.77	84	49.51	50.19
*P. putida* HB3267	CP003738.1	5.88	158	61.55	62.11
*P. putida* F1	CP000712.1	5.96	94	61.51	62.14
*P. putida* GB-1	CP000926.1	6.08	142	62.39	62.99

**Table 2:** Summary of T2-2 draft genome compared with other *P. putida* complete genomes available in RAST.

Sample of *P. putida *	Genome ID in RAST	Genome size (Mbp)	Total CDSs
T2-2	303.65	5.5	5053
F1	351746.4	6.0	5252
GB-1	76869.3	6.1	5410
W619	390235.3	5.8	5182

**Table 3:** Antibiogram of *Pseudomonas putida* strain T2-2. Antimicrobial compounds with MICs higher than expected were regarded as resistant by strain T2-2 and marked as “R” while the compounds with lower or equal to expected MICs were interpreted as sensitive for strain T2-2 and were marked as “S”.

University of Malaya					
BioMerieux customer:	Laboratory report			Printed on July 31, 2014, 20:42 SGT	
System number:
Printed by labsuper					
Isolate group: t2 2-1
Card type: AST-GN83 Testing Instrument: 000015F14C94 (Serial No. 4058)

Organism quantity:					
Selected organism: *Pseudomonas* spp.				

**Comments:**					

**Identification information**					

**Selected organism**	*Pseudomonas* spp.				
	**Entered:**	July 30, 2014, 14:35 SGT **By**: system			

**Analysis messages**:					
The following antibiotic(s) are suppressed from analysis: aztreonam

Susceptibility information	**Card**:	AST-GN83	**Lot number**: 673305210	**Expires: **	April 23, 2015 12:00 SGT
	**Completed**:	July 31, 2014, 06:17 SGT	**Status**: final	**Analysis time:**	15.50 hours

**Antimicrobial**	**MIC**	**Interpretation**	**Antimicrobial**	**MIC**	**Interpretation**

Ampicillin	>=32	R	Ceftriaxone	8	S
Amoxicillin/clavulanic acid	>=32	R	Cefepime	<=1	S
Ampicillin/sulbactam	>=32	R	Aztreonam		
Piperacillin/tazobactam	8	S	Meropenem	4	S
Cefazolin	>=64	R	Amikacin	<=2	S
Cefuroxime	>=64	R	Gentamicin	<=1	S
Cefuroxime axetil	>=64	R	Ciprofloxacin	0.5	S
Cefoxitin	>=64	R	Nitrofurantoin	>=512	R
Cefotaxime	8	S	Trimethoprim/sulfamethoxazole	>=320	R
Ceftazidime	2	S			

+ = deduced drug, ∗ = AES modified, and ∗∗ = user modified		

AES findings:	**Last modified**: May 28, 2014, 14:17 SGT			**Parameter set:**	global CLSI-based+natural resistance

**Confidence level**: analysis not performed			

**Action**	**Name (user ID)**		**Date/time**	**Comment**	
**Reviewed by:**	**labsuper**		**July 31**, **2014, 14:07 SGT**

Installed VITEK 2 systems version: 07.01.

MIC interpretation guideline: global CLSI-based.   Therapeutic interpretation guideline: natural resistance.

AES parameter set name: global CLSI-based+natural resistance.   AES parameter last modified: May 28, 2014, 14:17 SGT.
