# Supplementary material for: Global Genome Comparative Analysis Reveals Insights of Resistome and Life-Style Adaptation of Pseudomonas putida Strain T2-2 in Oral Cavity
Source: ScientificWorldJournal. 2014 Nov 10;2014:930727. doi: 10.1155/2014/930727 (PMC4243125; doi:10.1155/2014/930727)
Supplement: Supplementary file 1 — Pseudomonas putida strain T2-2 carries genes that encode for multidrug resistance efflux pump as well as multidrug and toxin extrusion family efflux pump. These genes were not found within the environmental Pseudomonas (reference genome from RAST).The presence of these efflux pumps protect it from the antibiotic and antimicrobial compounds which were presence in the human oral cavity. [file 930727.f1.pdf]

Supplementary Table 1: Multidrug resistance efflux pump genes identified in T2-2 genome annotation in RAST and their percentage homology with the closest respective gene

Multidrug resistance efflux pump genes		Start	End	Size (nt)	Contig	% homology with closest respective gene in RAST
RND efflux system	Membrane fusion protein CmeA	7033	8175	1143	5	*P. fluorescens* SBW25 (70%)
	Inner membrane transporter CmeB	8187	11312	3126	5	*Pseudomonas fluorescens* SBW25(80%)
	Inner membrane transporter CmeB	6806	3654	3153	195	*Pseudomonas fluorescens* SBW25(80%)
	Outer membrane lipoprotein CmeC	3657	2203	1455	195	*Pseudomonas fluorescens* SBW25(78%)
	Outer membrane lipoprotein CmeC	28389	26965	1425	42	*Salmonella enterica* subsp. enterica serovar Choleraesuis str. SC-B67 (70%)
	Outer membrane lipoprotein CmeC	13706	12339	1368	112	*Ralstonia eutropha* JMP134 (43%)
	Type I secretion outer membrane protein, TolC precursor	9874	11310	1437	155	*Pseudomonas fluorescens* SBW25(82%)
	Type I secretion outer membrane protein, TolC precursor	21693	23075	1383	22	*Azotobacter vinelandii* (61%)
Multidrug and toxin extrusion (MATE) family efflux pump YdhE/NorM, homolog		1119	2507	1389	210	*Pseudomonas fluorescens* SBW25 (75%)

Membrane fusion protein of RND family multidrug efflux pump	7964	6810	1155	195	*Pseudomonas fluorescens* SBW25 (77%)
RND multidrug efflux transporter; Acriflavin resistance protein	14627	11583	3045	191	*Pseudomonas fluorescens* SBW25 (85%)
RND multidrug efflux transporter; Acriflavin resistance protein	31667	28491	3177	42	*Pseudomonas aeruginosa* PAO1 (72%)
